# Crosstalk Between microRNAs and the Pathological Features of Secondary Lymphedema

**DOI:** 10.3389/fcell.2021.732415

**Published:** 2021-10-18

**Authors:** Khairunnisa’ Md Yusof, Kira Groen, Rozita Rosli, Kelly A. Avery-Kiejda

**Affiliations:** ^1^Hunter Medical Research Institute, New Lambton Heights, NSW, Australia; ^2^School of Biomedical Sciences and Pharmacy, College of Health, Medicine and Wellbeing, The University of Newcastle, Newcastle, NSW, Australia; ^3^Department of Biomedical Sciences, Faculty of Medicine and Health Sciences, Universiti Putra Malaysia, Serdang, Malaysia; ^4^UPM-MAKNA Cancer Research Laboratory, Institute of Bioscience, Universiti Putra Malaysia, Serdang, Malaysia

**Keywords:** microRNA, lymphatics, lymphangiogenesis, fibrosis, inflammation, lymphedema, immune dysfunction

## Abstract

Secondary lymphedema is characterized by lymphatic fluid retention and subsequent tissue swelling in one or both limbs that can lead to decreased quality of life. It often arises after loss, obstruction, or blockage of lymphatic vessels due to multifactorial modalities, such as lymphatic insults after surgery, immune system dysfunction, deposition of fat that compresses the lymphatic capillaries, fibrosis, and inflammation. Although secondary lymphedema is often associated with breast cancer, the condition can occur in patients with any type of cancer that requires lymphadenectomy such as gynecological, genitourinary, or head and neck cancers. MicroRNAs demonstrate pivotal roles in regulating gene expression in biological processes such as lymphangiogenesis, angiogenesis, modulation of the immune system, and oxidative stress. MicroRNA profiling has led to the discovery of the molecular mechanisms involved in the pathophysiology of auto-immune, inflammation-related, and metabolic diseases. Although the role of microRNAs in regulating secondary lymphedema is yet to be elucidated, the crosstalk between microRNAs and molecular factors involved in the pathological features of lymphedema, such as skin fibrosis, inflammation, immune dysregulation, and aberrant lipid metabolism have been demonstrated in several studies. MicroRNAs have the potential to serve as biomarkers for diseases and elucidation of their roles in lymphedema can provide a better understanding or new insights of the mechanisms underlying this debilitating condition.

## Introduction

Lymphedema is a serious chronic condition characterized by swelling, resulting from the abnormal accumulation of protein-rich lymph fluid in the interstitial spaces due to an imbalance between lymph fluid production and transport (Greene and Maclellan, 2013; Ducoli and Detmar, 2021). Unlike primary lymphedema, which is usually driven by inherited mutations, secondary lymphedema is a consequence of cancer treatment (i.e., radiotherapy or surgery) or infections. Radiotherapy and surgical excision induce trauma or insults to the lymph nodes and lymphatic structures, leading to the obstruction of lymph flow and the accumulation of protein-rich fluid at the affected area (Cueni and Detmar, 2008; Alitalo, 2011). Prolonged blockage of lymphatic flow and the accumulation of lymph lead to the pathological features of lymphedema, including inflammation, immune dysfunction, tissue remodeling, fibrosis, and aberrant lipid metabolism. To date, there is no molecular-based therapy for secondary lymphedema and the condition is usually treated with massage, manual lymphatic drainage, compression bandages, remedial exercise, and dietary intervention programs (Do et al., 2017; Jung et al., 2020). Although, the damage to lymphatic vessels may be treated through pharmaceutical therapies, stimulating lymphangiogenesis; clinical trials to evaluate growth factor therapy have yet to be conducted (Forte et al., 2019). In the absence of targeted therapies, further investigations into the molecular mechanisms of lymphedema may highlight additional treatment avenues. The aim of this review is to identify the epigenetic mechanisms, including small regulatory RNAs, which are aberrant in the processes that cause this condition, this may highlight dysregulated pathways in lymphedema and reveal novel therapeutic targets.

Hereditary or primary lymphedema is associated with mutations in genes that encode for lymphatic endothelial markers; vascular endothelial growth factor-C (VEGFC) and its receptor (VEGFR), such as Fms-related receptor tyrosine kinase-4 (*FLT4)*, SRY-box transcription factor 18 *(SOX18)*, forkhead box C2 (*FOXC2*), and angiopoietin (*ANGPT2)* (Miaskowski et al., 2013; Brouillard et al., 2014; Leppanen et al., 2020). Meanwhile, studies on genetic predisposition of secondary lymphedema have demonstrated a significant association of *VEGFR*, RAR-related orphan receptor C (*RORC*), *FOXC2*, and interleukin-6 (*IL6*) genes with secondary lymphedema (Newman et al., 2012; Miaskowski et al., 2013; Leung et al., 2014). However, the reported genes explain <30% of this debilitating condition (Leppanen et al., 2020), hence more studies are needed to further clarify the pathways involved in lymphedema. Epigenetics can alter gene expression in the absence of genomic mutations and may contribute to the pathophysiology of lymphedema. One form of epigenetics that has gained increasing interest over the years due to its biomarker potential and possible involvement in post-transcriptional gene regulation, are microRNAs (miRNAs) (Alitalo, 2011; Treiber et al., 2019). MiRNAs are non-coding RNAs of 20–25 nucleotides and their biogenesis involves transcription of larger primary miRNAs by polymerase II, cleavage by the nuclear enzyme Drosha into pre-miRNAs, and then export into the cytoplasm through exportin 5. In the cytoplasm, pre-miRNAs undergo a final processing step orchestrated by Dicer, to form mature miRNAs. Mature miRNAs can bind to the 3′untranslated region (3′UTR) of messenger RNA (mRNA) causing translational repression or degradation through the RNA-induced silencing complex (RISC) (Treiber et al., 2019). MiRNA expression has been linked to a plethora of physiological processes and their dysregulation has been linked to many diseases, sparking the development of therapeutic miRNA inhibitors (Baumann and Winkler, 2014). Many miRNA functions were elucidated through knockout and overexpression models, and tools to predict their target genes based on sequence complementarity to 3′UTRs have also been developed and are vital for hypothesis generation (Chen et al., 2019). The regulation of miRNAs has been extensively reviewed by several research groups (Roberts, 2014; Liu et al., 2018; Paul et al., 2018). MiRNAs represent an attractive non-invasive biomarker for diseases including lymphedema, because they are stable and detectable in bodily fluids such as serum, plasma, and urine (Babalola et al., 2013). This review will elucidate the roles of miRNAs in the pathological features of secondary lymphedema including inflammation, immune dysfunction, formation of fibrous tissue, and obesity (Escobedo and Oliver, 2017; Kataru et al., 2019b; Yuan et al., 2019; Azhar et al., 2020).

## miRNA Regulation in Pathological Features of Secondary Lymphedema

**FIGURE 1:**
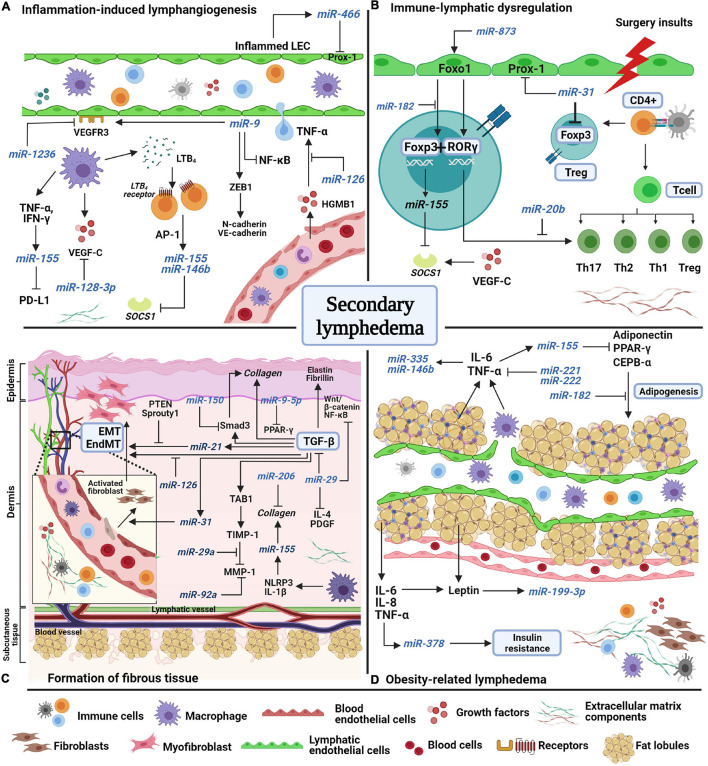
A schematic model of miRNAs’ potential involvement in the pathological features of secondary lymphedema. **(A)** Inflammation-induced lymphangiogenesis involves the release of inflammatory factors from both lymphatic and blood endothelial cells. Lymphatic markers, VEGF-C and VEGFR-3 are negatively regulated by miR-1236, miR-128-3p, and miR-9. miR-466, miR-155, and miR-146b are expressed during inflammation to induce lymphangiogenesis. **(B)** Surgery insults to lymphatic vessels and lymph nodes induces abnormal immune regulation as immune cells are trafficked in lymphatic vessels. Due to the disruption of lymph flow, immune cells accumulate in the extracellular matrix (ECM) and start to proliferate and differentiate into regulatory (Treg) and T helper cells, which produce inflammatory factors. Unlike miR-155, miR-31 and miR-182 negatively regulate Foxp3 expression to suppress Treg cell differentiation. Foxo1 induces Foxp3 and Rorγ expression, but the interaction is halted by miR-182 and miR-20b. **(C)** The formation of fibrous tissue of the skin occurs over time as ECM components increase excessively in the dermis and subcutaneous layers. The main factor of fibrosis, transforming growth factor-beta (TGF-β), mediates collagen fiber production, fibroblast differentiation, and endothelial and epithelial-mesenchymal transformation (EndMT/EMT) by directly regulating pro-fibrotic factors (TAB1, Smad3) and pro-fibrotic miRNAs such as mir-31, miR-21, and miR-155. MiRNAs such as miR-29a, miR-150, miR-9-5p, miR-126, and miR-92 act as anti-fibrotic miRNAs by suppressing the expression of fibrotic factors in multiple signaling pathways. **(D)** Obesity increases the risk of secondary lymphedema due to excessive accumulation of fat lobules that compress the lymphatic vessels, resulting in disruption of lymph flow. Adipokines and inflammatory factors induce the expression of miR-378, miR-199a-3p, miR-221, miR-222, miR-146b, and miR-335 which facilitate insulin resistance and fat expansion. Several miRNAs act to combat adipogenesis, such as miR-155 and miR-182. AP-1, activating protein-1; CCAAT-enhancer binding protein alpha, CEPB-α; chemokine C-C motif ligand-21, CCL21; cluster of differentiation 4 cells, CD4 +; regulatory T cell, Treg; endothelial-mesenchymal transformation, EndMT; epithelial-mesenchymal transformation, EMT; forkhead box O1, Foxo1; forkhead box P-3, Foxp3; high mobility group box-1 HMGB-1; interferon-gamma, IFN-γ; interleukin, IL; LTB_4_, leukotriene-B4; matrix metalloproteinase-1, MMP1; nuclear factor kappa-light-chain-enhancer of activated B cells, NF-κB; NOD-like receptor protein-3, NLRP3; peroxisome proliferator-activated receptor gamma, PPAR-γ; phosphatase and tensin homolog, PTEN; platelet-derived growth factor, PDGF; programmed death-ligand-1 PD-L1; prospero-homeobox 1 Prox-1; RAR-related orphan receptor gamma, ROR-γ; suppressor of cytokine signaling-1 SOCS1; TGF-beta activated kinase 1, TAB1; T helper cell, Th; TIMP metallopeptidase inhibitor 1, TIMP1; tumor necrosis factor-alpha, TNF-α; vascular endothelial growth factor receptor-3, VEGFR3; zinc finger E-box binding homeobox-1, ZEB1.

Over the past decade, correlative studies on animal models and human revealed several factors that lead to the development of secondary lymphedema. Although research into the regulation of miRNAs in secondary lymphedema is still in its infancy, several miRNAs have been identified and implicated in inflammation, immune system dysregulation, fibrosis, and obesity, all of which play a role in lymphedema (Alitalo, 2011; García Nores et al., 2016; Kataru et al., 2019b; Azhar et al., 2020). A summary of the interplay between miRNAs and factors associated with secondary lymphedema is presented in Figure 1.

## miRNAs and Inflammation-Induced Lymphangiogenesis

Mounting evidence suggests that lymphatic injury caused by radiotherapy or surgical intrusion results in chronic inflammatory changes (Kataru et al., 2019a; Allam et al., 2020). The earliest factors that are activated in lymphatic injury are danger-associated molecular patterns (DAMPs), endogenous cellular products that induce a pro-inflammatory state in the damaged tissue (Kataru et al., 2019a). Danger-associated molecular patterns are expressed in lymphatic endothelial cells (LECs), blood endothelial cells (BECs), and adipocytes. Two common DAMPs, high mobility group box 1 (HMGB1) and heat shock protein 70 (HSP-70), were found to be highly expressed in mouse tail-lymphedema tissues and 5-mm punch biopsies of human lymphedematous tissue compared to unaffected sites (Zampell et al., 2011, 2012a). HMGB1 was also shown to regulate lymphangiogenesis *in vivo* and its blockage resulted in inflammatory lymphangiogenesis suppression (Qiu et al., 2012; Kataru et al., 2019a). A recent study by Tang et al. (2017) demonstrated *HMGB1* harbors a miR-126 binding site in its 3′UTR. Under the hyperglycemic conditions, miR-126 targeted *HMGB1* and remarkably attenuated HMGB1 protein expression in human umbilical vein endothelial cells (HUVEC), via Akt/eNOS (endothelial nitric oxide synthase) signaling. Subsequently, the miR-126/HMGB1 interaction suppressed downstream elements of HMGB1, including tumor necrosis factor-α (TNF-α) and nicotinamide adenine dinucleotide phosphate oxidase (NADPH oxidase) (Tang et al., 2017).

Lymphatic stasis induces the accumulation of immune cells including macrophages, which promote VEGF-C/VEGFR signaling, a main factor in lymphatic vessel development and inflammation-induced lymphangiogenesis (Ogata et al., 2016; Kataru et al., 2019a). Although VEGF-C is critical in normal lymphatic development, a recent study has reported that VEGF-C promotes the pathogenesis of lymphedema by instigating lymphatic leakage through VEGFR-2 (Gousopoulos et al., 2017). A study by Ogata et al. demonstrated that initial and active lymphangiogenesis is essential for development of chronic lymphedema as evidenced by increased neolymphatic vessels that remained dilated after three months of lymphatic obstructions in a mouse model (Ogata et al., 2016). The expression of VEGF-C in the edematous tissue was increased after four days of lymphatic obstruction and inhibition of VEGFR-3 using VEGFR3-Fc competitor protein suppressed the generation of small lymphatic vessels and histological features of lymphedema, including fibrosis and adipogenesis (Ogata et al., 2016). Moreover, VEGF-C was found to be increased in the serum of breast cancer-related lymphedema patients (Ghanta et al., 2015; Jensen et al., 2015) and the edematous tail-lymphedema tissues of a mouse model (Rutkowski et al., 2006). Jones et al. reported that miR-1236 could negatively regulate VEGFR-3 expression as well as its associated pathways including Akt and extracellular signal-regulated kinase 1/2 (ERK1/2) to alleviate migration and proliferation of human LECs *in vitro* (Huggenberger et al., 2011; Jones et al., 2012). In another study, miR-128-3p was found to interact directly with the 3′UTR of *VEGF-C* and *VEGFR-3*, suppressing the proliferation of LECs, through Ca^2+^ and ERK1/2 signaling in a VEGF-C concentration dependent manner (Zhou et al., 2018).VEGFR-3 was also reported to be regulated by miR-9, thereby promoting the lymphangiogenesis through LEC proliferation within the mesenteric lymphatic vessels of rats (Chakraborty et al., 2015). miR-9 acts by directly targeting *NFKB1* and activating VEGFR-3 in LECs. In addition, miR-9 induced expression of pro-lymphangiogenic factors such as zinc finger E-box binding 1 (ZEB1), N-cadherin and VE-cadherin (Chakraborty et al., 2015).

In line with the induction of inflammation during lymph stasis, macrophages also released a large amount of leukotriene B_4_ (LTB_4_), an activator of leukocytes and a potent lipid chemotactic factor for neutrophils (Tian et al., 2017; Jiang et al., 2018). Tian et al. reported that LTB_4_ promotes lymphedema development in post-surgical lymphedema (Tian et al., 2017). At low concentrations (10nM), LTB_4_ demonstrated pro-lymphangiogenic capacity but at higher concentrations (400 ηM), LTB_4_ was found to inhibit Notch signaling in LEC, an important pathway for lymphatic maintenance and development (Murtomaki et al., 2014; Tian et al., 2017). Further analysis showed that blockade of LTB_4_ signaling decreases macrophages, CD4 + T cell and neutrophil infiltration into the lymphedematous tissue. Additionally, LTB_4_ concentration in serum is significantly elevated in lymphedema patients (Tian et al., 2017). Due to encouraging findings, a new drug, Ubenimex is designed and intended to benefit lymphedema patients through inhibition of LTB_4_. The efficacy of Ubenimex has been documented in an experimental model (Tian et al., 2017; Cribb et al., 2021) and is currently the subject of a clinical trial (NCT02700529) (Rockson et al., 2018). A study by Wang et al. reported activation of LTB_4_ and its receptor, B-leukotriene receptor-1 (BLT1) enhanced the expression of inflammatory miRNA, miR-155 and miR-146b through transcription factor, activating protein (AP-1) in leukotriene-deficient mice (Wang et al., 2014). The two miRNAs further target the suppressor of cytokine signaling-1 (SOCS-1) to induce its degradation, and subsequently enhance macrophage activation (Wang et al., 2014).

Besides VEGF-C,VEGFR-3, and LTB_4_, infiltration of macrophages into the affected sites promotes the expression of cytokines and inflammatory factors including interleukins (IL-4, IL-6, IL-10), TNF-α, interferon-γ (IFN-γ), and transforming-growth factor-beta (TGF-β) in lymphedema studies (Lin et al., 2012; Miaskowski et al., 2013; Leung et al., 2014). TNF-α and IFN-γ were reported to synergistically induce miR-155 expression in inflamed human dermal LECs and human dermal fibroblasts. The induction resulted in the suppression of the downstream adaptive immune factor, programmed death ligand-1 (PD-L1) (Yee et al., 2017). In another study, miR-466 was reported to bind to the 3′UTR of prospero-related homeobox-1 (*PROX1*) and inhibited proliferation of LECs in a corneal burn alkali animal model (Seo et al., 2015). Prox-1 is a major transcription factor of lymphatic development and mutations of the *PROX1* gene have been reported in lymphedema patients (Ricci et al., 2020). Studies on the effects of *PROX1* knockout in mouse models induced lymphatic vasculature dysfunction (Srinivasan and Oliver, 2011; Bui and Hong, 2020). Studies on the role of miRNAs in lymphedema-related inflammation are still lacking and some of the reported miRNAs have yet to be validated in human samples, using different diseases and models. A simplified mechanism of miRNA’s-roles in inflammation is illustrated in Figure 1A.

## miRNAs and Immune-Lymphatic Dysregulation

Accumulation of lymph fluid promotes infiltration of immune cells to the affected limbs. One of the major hallmarks of lymphedema is the increase of CD4^+^ T-cells and regulatory T cells (Tregs) in lymphedematous tissue of lymphedema patients and mouse models (García Nores et al., 2018). It was reported that infiltration of CD4^+^ T cells (Th1, Th2, Th17) was increased after the axillary lymph node excision and Tregs proliferation was prominent at the distal tissue of the lymphatic injury (Ogata et al., 2016; García Nores et al., 2018). Depletion of CD4^+^ T cells using CD4^+^ specific antibodies reduced tail volume and tissue area covered by the lymphatic vessels after four weeks of surgery. Moreover, the treatment increased the amount of PEGylated NIR dye reaching the lymphatic area, indicating increased lymphatic vascular networks (Proulx et al., 2013; Gousopoulos et al., 2016). Tregs are characterized by the expression of forkhead box P3 (Foxp3) that controls Treg development and function. Notably, Foxp3^+^ and CD45^+^ cells were elevated in skin biopsies of lymphedema patients and adoptive Tregs were found to reduce tail edema in mice after surgery (Gousopoulos et al., 2016).

To our knowledge, regulation of miRNAs in immune cell signaling in lymphedema has never been reported. However, findings from several studies might be relevant to the pathological condition of lymphedema (Figure 1B). For instance, Foxp3 was found to positively regulate miR-155 and the deletion of miR-155 resulted in the depletion of Tregs in the thymus and periphery of mice (O’Connell et al., 2010; Lu et al., 2015). miR-155 was reported to target SOCS1, a negative regulator of IL-2. Foxp3 drives the elevation of miR-155 thus reduced SOCS1 protein expression, heightened the sensitivity of Treg cells to IL-2, and led to an increase of Tregs numbers (Lu et al., 2009, 2015). In an infection-induced lymphedema study, SOCS1 was identified to be regulated by VEGF-C/VEGFR-3 and its expression inhibited the Toll-like receptor (TLR)-NF-κB signaling, an important pathway that prevents uncontrolled inflammation during bacterial infection (Zhang et al., 2014).

A transcription factor of Th17, RAR-related orphan receptor-gamma (Rorγ) has been reported to interact with *FOXP3* to induce Treg cell differentiation during exposure to IL-6 and IL-21. In contrast, Foxp3 is released from Rorγ in the presence of the anti-inflammatory factors, inducing the differentiation of Th17 cells (Zhou et al., 2008). miR-20b was identified to target *RORC* and decrease Th17 cell differentiation in experimental autoimmune encephalomyelitis (Pan et al., 2015). Although the results of this study were reported in an autoimmune disease model, the Rorγ protein is encoded by *RORC*, a gene that was highly correlated with lymph node organogenesis (Massoud et al., 2016), filariasis, (Rajasekaram et al., 2017) and secondary lymphedema (Newman et al., 2012; Michelini et al., 2020). A LEC marker, forkhead box O1 (Foxo1), was demonstrated to interact with Rorγ, resulting in the regulation of Th17 cell pathogenicity and IL-17A production. miR-873 was reported to target *FOXO1* and facilitate Th17 cell differentiation in systemic lupus erythematosus patients (Liu et al., 2017). Foxo1 plays an essential role in Foxp3 expression in Tregs through a Foxo1/miR-182 dependent pathway. Foxo1 mediates the downregulation of miR-182 and increases the proportion of Foxp3 cells in the peripheral lymph nodes and spleen of mice with acute autoimmune encephalomyelitis (Wan et al., 2016). The contribution of Foxo1 and miR-182 to immune-related disease reflect their reciprocal interaction in lymphatic vascular development (Chen et al., 2016; Niimi et al., 2020), suggesting that these two factors may play a role in lymphatic-related diseases. Additionally, an *ex vivo* study on human Tregs found that miR-31 binds to the 3′UTR of *FOXP3* and negatively regulates Foxp3 expression. The transfection of anti-miR-31 into Treg cells increased Foxp3 expression by 14-fold (Rouas et al., 2009). Interestingly, miR-31 is also a negative regulator of normal lymphatic development by acting on LEC signature genes such as *PROX1* and *FOXC2* (Pedrioli et al., 2010). Collectively, it would be of great interest to further validate these miRNA findings in lymphedema, as some miRNAs (i.e., miR-31 and miR-182) may play an overlapping role in immune response regulation and lymphatic development.

## miRNAs and the Formation of Fibrotic Tissues

One of the hallmarks of secondary lymphedema is fibrosis, which is characterized by the deposition of extracellular matrix (ECM) proteins in the dermis and subcutaneous tissue, leading to hardening, inflexibility, and non-pitting edema with a *peau d’orange* look on the skin. Increased amounts of collagen fibers in the edematous skin were found in lymphedema patients (Gardenier et al., 2016) and animal models (Zampell et al., 2012b). Key processes involved in tissue fibrosis are TGF-β signaling, synthesis of ECM molecules, and fibroblast differentiation. TGF-β signaling plays a pivotal role in accelerating fibrosis by regulating profibrotic factors such as collagen, laminin, fibronectin, and elastin (Figure 1C).

miR-29 has been reported to be a negative regulator of skin fibrosis by targeting profibrotic proteins; TGF-β, platelet-derived growth factor (PDGF), and IL-4 (Maurer et al., 2010). Negative regulation of miR-29 and miR-206 reduced the expression of type I, type VI, and type XXIX collagen in scleroderma (Li et al., 2012; Peng et al., 2012). Apart from collagen, miR-29 was found to inhibit the synthesis of elastin and fibrillin by regulating *TGF-*β, and other profibrotic pathways, including Wnt/beta catenin, NF-kB, and mitotic-activated protein kinase (MAPK) (Peng et al., 2012). Further, the interaction of miR-29a with TGF-β activated kinase binding protein-1 (*TAB1*) inhibited the expression of tissue inhibitor of metalloproteinases-1 (TIMP-1), and increased the action of matrix metalloproteinase (MMP-1), an enzyme that breaks down the ECM (Ciechomska et al., 2015; O’Reilly, 2016).

Contrastingly, miR-92a, which was highly expressed in the serum and skin fibroblast of scleroderma patients, was found to contribute to MMP-1 downregulation (Sing et al., 2012). Downregulation of miR-150 induced phosphorylation of Smad3 and activation of TGF-β signaling, subsequently increasing the transcription of collagen in skin fibrosis. These findings are in line with elevated miR-150 levels in the serum of scleroderma patients correlating with thicker skin (Honda et al., 2013). Although the role of miRNAs in fibrosis in lymphedema has yet to be elucidated, TGF-β plays significant role in lymphedema (Oka et al., 2008; Vittet et al., 2012). Lin et al. reported significant upregulation of TGF-β expression in skin punch biopsies compared to paired normal tissue of lymphedema patients. The elevated expression of TGF-β was also reflected in the serum of lymphedema patients and pathway analysis revealed involvement of TGF-β in chronic lymphedema hallmarks namely fibrosis, dermal and epidermal cellular growth (Lin et al., 2012). An *in vivo* study demonstrated that Smad-mediated activation of TGF-β1 from infiltrating macrophages leads to the transition of fibroblasts to myofibroblasts. This process occurs during the acute to subacute phase of lymphedema (Sano et al., 2020). The crosstalk between miRNAs and TGF-β in endothelial-mesenchymal transformation (endMT) and EMT have been by reported in some studies. For instance, miR-31 positively regulates expression of mesenchymal markers during TGF-β-endMT in mouse MS-1 pancreatic microvascular endothelial cells (Katsura et al., 2016). miR-126 has been demonstrated to inhibit endMT through phosphoinositide-3-kinases PI3K/Akt/Smad4 signaling by directly targeting PI3K-receptor 2 (*PI3KR2*) in endothelial progenitor cells. Moreover, deficiency of miR-126 leads to the induction of endMT by TGF-β in TGF-β1-treated endothelial progenitor cells (Zhang et al., 2013). Interestingly, the different roles of miR-31 and miR-126 in regulating TGF-β in endMT reflect their properties as anti- and pro-lymphangiogenic factors in developmental lymphangiogenesis (Md Yusof et al., 2020).

TGF-β signaling also induced the expression of miR-21, which in turn, targets Smad7 to reduce its expression and enhance the profibrotic effect of TGF-β. miR-21 was identified to be involved in EMT, by targeting tensin homolog (*PTEN*), an inhibitor of EMT (Romano and Schepis, 2012). Additionally, miR-21 increased fibroblast differentiation by downregulating the fibroblast differentiation inhibitor, *Sprouty1* through the ERK pathway (Kumarswamy et al., 2011). In a recent study on skin fibrosis, overexpression of miR-9-5p in TGF-β activated human dermal fibroblasts abrogated TGF-β signaling through modulation and silencing of *TGF*β*R2* (Miguel et al., 2016). miR-9-5p targets peroxisome proliferator activated-receptor gamma (*PPARG*) to downregulate ECM factors (α-smooth muscle, vimentin and collagen 1A) and induce apoptosis in hypertrophic scar fibroblasts (Chai et al., 2020). Besides being highly expressed during inflammation, miR-155 is elevated in fibrotic tissue and the lack of miR-155 expression abolishes fibrosis (Artlett et al., 2017). Induction of miR-155 through IL-1β and the inflammasome node like receptor protein-3 (NLRP3) signaling pathway, promotes collagen production (Artlett et al., 2017). Overall, the reported findings highlight the significant regulatory factors associated with fibrogenesis such as TGF-β, miR-29, miR-31, and miR-126. It is noteworthy that a new drug, Remlarsen (MRG-201), currently in Phase II clinical trials, is designed to mimic the activity of miR-29 to decrease the expression of collagen and the formation of keloid in subjects with a history of keloid scars (Gallant-Behm et al., 2019). Additionally, a newly developed inhibitor of miR-92a (MRG-110), is intended to accelerate wound healing by inhibiting the activation of myofibroblasts (Gallant-Behm et al., 2018). Perhaps, these drugs may provide beneficial effects by halting fibrogenesis in lymphedema patients too.

## miRNAs and Obesity-Related Lymphedema

Previous studies demonstrated a reciprocal relationship between obesity and post-operative lymphedema. It was reported that subcutaneous fat deposition and fat thickness increased after surgical excision of dermal lymphatic vessels (Aschen et al., 2012; Escobedo et al., 2016). The risk of secondary lymphedema increases in obesity due to the abnormal accumulation of fat lobules at the affected area and compression of lymphatic capillaries, leading to eventual disruption of fluid and lipid transport through the lymphatic system (García Nores et al., 2016; Azhar et al., 2020). On the other hand, injured lymphatic vessels from surgery can drive adipose deposition, which promotes proliferation of local adipose tissue and subsequently leads to low grade inflammation. Adipocyte proliferation is modulated by the upregulation of adipose differentiation genes, *PPARG* and CCAAT enhancer-binding protein alpha (*CEPBA)* (Aschen et al., 2012). Overexpression of transfected miR-155 decreased expression levels of adipogenic markers including PPAR-γ, CEPB-α, and adiponectin in 3T3-L1 adipocytes. Interestingly, further pathway analysis revealed that miR-155 overexpression resulted in increased inflammatory cytokine and chemokine expression (Liu et al., 2011; Karkeni et al., 2016). Overexpression of miR-182 in 3T3-L1 cells and human visceral adipocytes greatly inhibited adipocyte differentiation by directly targeting CEPB-α, suggesting miR-182 is a negative regulator of adipogenesis (Dong et al., 2020).

Pathological adipose remodeling also induced the dysregulation of adipokine production (leptin and adiponectin) by increasing the release of inflammatory factors, including TNF-α, IL-6, IL-8, and monocyte chemoattractant protein (MCP-1). Increased levels of IL-6 (Cuzzone et al., 2014; Sato et al., 2016) and leptin (Sato et al., 2016; Zaleska and Olszewski, 2017) were observed in the serum of obese-lymphedema patients, probably reflecting the expansion of adipose tissue. Treatment of human LECs with high concentrations of leptin resulted in disorganization and morphological changes in lymphatic ducts and inhibited tube formation (Sato et al., 2016). Elevated miR-199a-3p expression was observed in mature human adipocytes compared to preadipocyte deposits from obese patients (Gu et al., 2016). The expression of miR-199a-3p was significantly induced by leptin, IL-6, and TNF-α. Interestingly, TNF-α, IL-6, and leptin induced the expression of miR-378 to promote lipogenesis in human adipocytes and led to insulin resistance (Gerin et al., 2010; Xu et al., 2014). An anti-miR-378 (MGN-5804) has been developed to regulate insulin resistance in metabolic disease (Carrer et al., 2012). The preliminary findings revealed that knock-out miR-378 mice were protected against diet-induced obesity and exhibit a reduction in adipocyte size (Carrer et al., 2012). Treatment of human adipocytes with leptin, resistin, IL-6, and TNF-α upregulated miR-335, which is encoded by the second intron of mesoderm-specific transcript (*MEST*), a gene involved in fat expansion (Zhu et al., 2014). Additionally, upregulation of IL-6 and TNF-α increased miR-146b expression in human mature adipocytes as a response toward obesity-related inflammation (Shi et al., 2014). Other miRNAs that target *TNFA* are miR-221 and miR-222, which were upregulated in muscle and liver tissue of obese rats (Chartoumpekis et al., 2012; Lustig et al., 2014). Levels of miR-221 in adipose tissue were positively correlated with higher body mass index, glucose and insulin concentration in the serum of obese patients (Meerson et al., 2013). Moreover, miR-221 may contribute to the development of insulin resistance, by affecting PPAR signaling pathways and directly downregulating *ETS1* and *ADIPOR1*. Meanwhile, miR-222 expression was significantly higher in plasma of obese patients and dropped in response to insulin administration. Thus, miR-222 may play a significant role in adipogenesis and insulin resistance (Vickers et al., 2011; Ortega et al., 2014). Taken together, these findings deepen the understanding that lymphedema is associated with abnormal metabolic mechanisms such as insulin resistance and overexpression of adipokines (Figure 1D). The elucidation of miRNA roles in obesity-related lymphedema may provide novel treatment targets.

## Conclusion and Future Perspectives

This review has highlighted several miRNAs that are involved in processes that contribute to secondary lymphedema. Most of the studies on miRNAs were conducted in autoimmune and inflammation-related diseases, suggesting that molecular investigation of secondary lymphedema is still lacking. Given that secondary lymphedema involves multiple events and has different stages, extensive studies are warranted to refine the characterization of miRNAs in lymphedema patients or animal models to provide new insights into the mechanisms underlying lymphedema and subsequently facilitate the development of molecular-based therapies for this condition.

To date, there are no FDA-approved drugs specifically designed to target lymphangiogenesis or lymphedema. The search for molecular-based therapies for lymphedema is still ongoing. There are limited studies on therapeutic interventions for lymphedema, highlighting the need for a different group of targets and miRNAs that could be of potential benefit in this area. Most of the reported studies on miRNA-based therapies are mainly for diseases such as cancer, hepatitis, and skin lesion (Hanna et al., 2019; Chakraborty et al., 2021). The development of potential drugs for lymphedema that target lymphatic vasculature may involve delicate work given that lymphangiogenesis actively occurs in metastatic cancers (Christiansen and Detmar, 2011; Md Yusof et al., 2020). Although interfering with lymphangiogenesis may reduce tumor size, it may also lead to the accumulation of lymph fluid in the affected area, hence necessitating new therapies to balance both issues carefully. We hypothesize that miR-126 could be exploited as a new approach for lymphedema therapy due to its function in lymphatic maturation and processes that contribute to lymphedema. Of importance, miR-126 has been shown to act as a tumor-suppressor miRNA in various cancers (Dong et al., 2016). Likewise, miR-31, a negative regulator of lymphatic vessels and an oncogenic miRNA (Yu et al., 2018), could also be a therapeutic target. Theoretically, inhibition of miR-31 should induce lymphagiogenesis and disrupt the expansion of tumor growth (Pedrioli et al., 2010; Yu et al., 2018). Taken together, these miRNA candidates may serve as potential dual-effect agents worth validating in lymphedema studies.

Undoubtedly, the field of miRNAs and biomarkers is promising but dealing with a multitude of effects of a miRNA remains a challenge in miRNA-based therapy. Emerging miRNA studies in different disease models suggests that while the dysregulation of miRNAs may be disease–specific, the effects of miRNA modulation may lead to undesirable off-target effects on normal cells or tissues (Rupaimoole and Slack, 2017; Chakraborty et al., 2021). Moreover, some miRNA delivery vehicles such as viral-based vector (adeno-associated virus) and polymer-based (polyethylamines, PEI) were reported to cause a strong immune response (Geisler and Fechner, 2016) and cytotoxicity to normal cells (Bai et al., 2019; Segal and Slack, 2020). It is well-documented that dysregulation of the immune system occurs in lymphedema patients (García Nores et al., 2018; Kataru et al., 2019a) and immune-related adverse effects may be fatal in the affected individuals. Therefore, more effective delivery systems need to be explored to improve the target-specificity and localized delivery of miRNA-based therapies in lymphedema patients.

## Author Contributions

KMY conceived the topic, outlined the manuscript, created the image, and wrote the review. KG co-wrote and edited the review. RR revised the review. KAA-K revised and edited the review. All authors reviewed the manuscript.

## Conflict of Interest

The authors declare that the research was conducted in the absence of any commercial or financial relationships that could be construed as a potential conflict of interest.

## Publisher’s Note

All claims expressed in this article are solely those of the authors and do not necessarily represent those of their affiliated organizations, or those of the publisher, the editors and the reviewers. Any product that may be evaluated in this article, or claim that may be made by its manufacturer, is not guaranteed or endorsed by the publisher.
